# Genetic diversity in *Eimeria* species infecting chickens: Implications for virulence, host immunity, vaccine performance, anticoccidial drug sensitivity and diagnostic reliability

**DOI:** 10.14202/vetworld.2026.2088-2104

**Published:** 2026-05-18

**Authors:** Nooran Mahomed Rafeer, Matthew Adekunle Adeleke

**Affiliations:** Discipline of Biological Sciences, School of Agriculture and Science, College of Agriculture, Engineering and Science, University of KwaZulu-Natal, Westville, South Africa

**Keywords:** anticoccidial resistance, chicken coccidiosis, diagnostic reliability, *Eimeria* diversity, genomic variation, immuno-genicity, vaccine efficacy, virulence

## Abstract

Avian coccidiosis, caused by multiple *Eimeria* species, remains a major challenge to global poultry production due to its impact on animal health, productivity, and economic sustainability. Increasing genomic and molecular evidence highlights substantial genetic diversity within and between *Eimeria* species, which plays a critical role in shaping parasite virulence, host immune responses, vaccine efficacy, sensitivity to anticoccidial drugs, and diagnostic accuracy. This review synthesizes current knowledge on the mechanisms and consequences of genetic variation in chicken-infecting *Eimeria* species. Variations such as single-nucleotide polymorphisms, insertions/deletions, and copy number changes influence key functional proteins involved in host invasion and immune evasion. Antigenic diversity contributes to incomplete cross-protection and variability in vaccine performance, while mutations in drug-target genes are associated with the emergence of resistance to commonly used anticoccidials. Additionally, genetic heterogeneity complicates conventional and molecular diagnostic approaches, leading to potential misidentification and underestimation of infection prevalence. Advances in whole-genome sequencing and high-throughput diagnostic tools provide opportunities to better understand parasite diversity and improve disease control strategies. Overall, integrating genomic insights with vaccination, drug management, and diagnostic innovations is essential for developing sustainable and effective coccidiosis control programs in poultry systems.

## INTRODUCTION

The poultry industry is one of the main suppliers of animal protein (meat and eggs) worldwide [1]. The United States Department of Agriculture reported that global chicken meat production reached 103.73 million metric tons in 2024–2025, representing a slight increase from 103.68 million metric tons in 2023 [2]. With the global human population projected to reach 9 billion by 2050, there is an urgent need to increase the production of safe, sustainable protein sources [3].

Coccidiosis is a major parasitic disease affecting the poultry industry worldwide and remains a critical constraint to efficient production. The disease is caused by infection with one or more species of *Eimeria*, which infect livestock, particularly chickens, across diverse production systems. Due to the infection pathway of *Eimeria* spp., the disease results in substantial economic losses and compromised animal welfare. It is estimated that avian coccidiosis leads to global losses of approximately US$3 billion annually [4]. *Eimeria* spp. are single-celled, obligate intracellular protozoan parasites belonging to the genus *Eimeria* within the phylum *Apicomplexa*. Infection occurs primarily in the intestinal tract, leading to a disease of considerable economic importance [5]. The genus comprises approximately 1800 species, among which *Eimeria brunetti*, *Eimeria maxima*, *Eimeria necatrix*, *Eimeria tenella*, *Eimeria acervulina*, *Eimeria mitis*, *Eimeria praecox*, *Eimeria zaria*, *Eimeria nagambie*, and *Eimeria lata* are the most common species infecting chickens [6]. Depending on the infecting species, disease severity ranges from subclinical infections to acute hemorrhagic enteritis associated with high morbidity and mortality [5].

The increasing availability of whole-genome sequences and advanced molecular tools has revealed extensive genetic diversity within and between *Eimeria* spp. The presence of single-nucleotide polymorphisms (SNPs), insertions/deletions, and copy number variations (CNVs) contributes to strain-specific phenotypic traits that influence parasite fitness and pathogenicity [7]. Genetic variation can alter the expression and structure of key virulence and immunogenic proteins, including surface antigens and apical complex proteins, thereby modifying host–parasite interactions and immune evasion mechanisms [8]. This diversity complicates vaccine development, as antigenic polymorphisms may reduce cross-protection among heterologous strains and geographically distinct isolates. Similarly, mutations in drug-target genes and transporter proteins have been associated with the emergence of resistance or altered sensitivity to commonly used anticoccidials [9].

In addition to affecting control strategies, genetic variation poses challenges for diagnostic accuracy. Conventional diagnostic approaches based on morphology, serology, or antigen detection may fail to distinguish closely related or co-infecting species. Molecular methods such as polymerase chain reaction (PCR), targeting sporozoite antigen genes, internal transcribed spacer (ITS) regions, or 18S ribosomal DNA (rDNA), have improved species-specific detection, particularly in mixed infections, enabling identification of minor species and field isolates [10]. However, genetic heterogeneity and polymorphisms within target regions can still lead to misidentification or underestimation of infection prevalence.

Collectively, these observations demonstrate that genetic variation in *Eimeria* spp. exerts interconnected effects on virulence, immunogenicity, vaccine performance, drug sensitivity, and diagnostic accuracy. These domains should therefore be considered as part of an integrated system rather than independent components. A comprehensive understanding of these interactions is essential for improving disease control strategies within a One Health framework.

Despite significant advances in genomics and molecular parasitology, several critical gaps remain in understanding the functional implications of genetic diversity in *Eimeria* spp. First, most studies have investigated virulence, immunogenicity, vaccine efficacy, drug resistance, and diagnostic performance independently, with limited integration across these domains. This fragmented approach restricts the ability to fully understand how genetic variation simultaneously influences multiple phenotypic traits. Second, although numerous polymorphisms, including SNPs and CNVs, have been identified, the majority of genotype–phenotype relationships remain inferred rather than experimentally validated, particularly under *in vivo* conditions. Third, current vaccine strategies often rely on a limited number of strains and may not adequately reflect the genetic diversity of field populations, leading to inconsistent protection. Fourth, diagnostic tools are frequently designed based on conserved loci but are not always validated across genetically diverse or geographically distinct populations, increasing the risk of false negatives or species misidentification. Finally, there is limited integration of genomic surveillance with practical control strategies, including vaccine updates, drug rotation, and field diagnostics, particularly in low- and middle-income poultry production systems.

**Figure 1:**
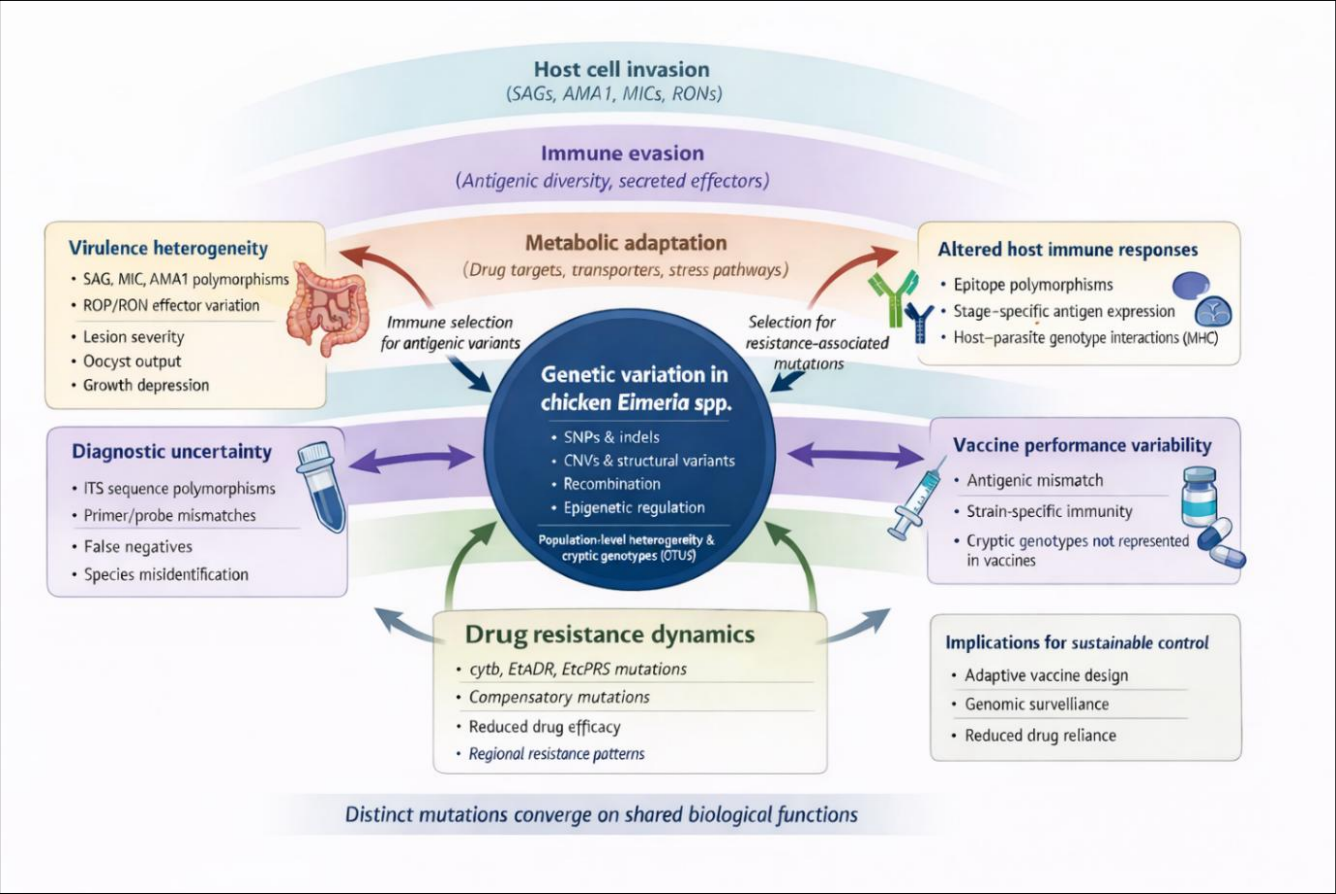
Genetic variation as the central driver of coccidiosis control failure and adaptation in chicken *Eimeria* spp.

This review aims to provide a comprehensive and integrated analysis of how genetic diversity within *Eimeria* spp. influences key phenotypic traits associated with coccidiosis in chickens. Specifically, the review seeks to (i) evaluate the role of genetic variation in shaping virulence and host immune responses, (ii) assess its impact on vaccine efficacy and cross-protection, (iii) examine the relationship between genomic mutations and anticoccidial drug sensitivity or resistance, and (iv) analyze how genetic heterogeneity affects the accuracy and reliability of current diagnostic approaches. Furthermore, this review aims to bridge the gap between genomic research and practical disease control by highlighting the importance of integrating genetic surveillance with vaccination strategies, drug management, and diagnostic innovations. By adopting a One Health perspective, the review ultimately aims to support the development of sustainable, evidence-based strategies for effective coccidiosis control in poultry production systems (Figure 1).

## REVIEW METHODOLOGY

### Ethical approval

This study was conducted as a narrative review and did not involve any *in vivo* or *in vitro* experimental procedures, animal handling, or human participation. Therefore, formal ethical approval from an institutional review board or animal ethics committee was not required. The review was performed in accordance with standard academic and scientific integrity guidelines, ensuring accurate representation, interpretation, and citation of previously published data. All included studies were sourced from peer-reviewed literature, and ethical compliance was assumed based on the original publications.

### Study design

This review was conducted as a narrative, evidence-based synthesis focusing on genetic variation in chicken-infecting *Eimeria* spp. and its implications for virulence, immunogenicity, vaccine efficacy, sensitivity to anticoccidial drugs, and diagnostic performance. The review was designed as an integrative synthesis that explicitly links genetic variation in *Eimeria* spp. to downstream phenotypic outcomes, rather than treating these domains as independent topics.

This review was conducted over a defined period corresponding to the literature search and analysis phase. The study did not involve a specific geographic sampling location, as it synthesized global research findings on *Eimeria* spp. infecting chickens. The literature included in this review comprises studies conducted across diverse geographic regions, thereby ensuring a broad and representative understanding of genetic variation, virulence, immunogenicity, vaccine efficacy, drug sensitivity, and diagnostic performance in different poultry production systems worldwide.

### Literature search strategy

A comprehensive literature search was performed using PubMed, Web of Science, Scopus, and Google Scholar. Searches employed combinations of keywords including *Eimeria*, chicken coccidiosis, genetic variation, whole-genome sequencing, SNPs, CNVs, virulence, immunogenicity, vaccine efficacy, anticoccidial resistance, drug sensitivity, molecular diagnosis, PCR, and next-generation sequencing. Boolean operators (“AND”, “OR”) were used to refine search results.

### Eligibility criteria

Peer-reviewed articles published primarily within the last two decades were included. Earlier studies were considered seminal, where relevant, for historical or conceptual context. Eligible studies included genomic and molecular investigations, experimental infection studies, vaccine and drug efficacy evaluations, and diagnostic assessments related to chicken-infecting *Eimeria* spp. Studies focusing exclusively on non-avian *Eimeria* spp. were excluded unless they provided relevant comparative insights.

### Study selection process

Titles and abstracts were initially screened for relevance, followed by full-text assessment of selected articles. Studies were included if they addressed genetic diversity, molecular or genomic variation, and their functional or applied consequences for pathogenicity, host immune responses, control strategies, or diagnostic accuracy.

### Data extraction and synthesis

Key information extracted included types of genetic variants (e.g., SNPs, insertions/deletions, CNVs, structural variants), affected genes or genomic regions, experimental models used, and reported phenotypic outcomes related to virulence, immunogenicity, vaccine protection, drug resistance, or diagnostic reliability. Due to heterogeneity in study designs and outcome measures, data were synthesized thematically rather than quantitatively.

### Data integration and interpretation

Findings were integrated across thematic sections to highlight mechanistic links between genetic variation and challenges in disease control, with emphasis on identifying research gaps, methodological limitations, and future directions for genomics-guided control strategies.

### Limitations

As a narrative review, this synthesis does not employ formal risk-of-bias assessment or quantitative meta-analysis, and conclusions are contingent on the scope and quality of available studies.

## CHICKEN COCCIDIOSIS

Avian coccidiosis is a severe parasitic disease caused by Eimeria spp., which belong to the phylum Apicomplexa. This infection occurs in the intestinal tract of chickens, resulting in growth impairment and immune suppression, thereby causing detrimental effects on animal health and increasing mortality rates [11, 12]. Globally, the burden of coccidiosis is disproportionately higher in low- and middle-income countries, where limited access to vaccines, anticoccidial drugs, biosecurity measures, and diagnostic infrastructure exacerbates disease prevalence and economic losses compared with high-income regions that employ intensive control strategies [13].

Chicken coccidiosis is prevalent wherever poultry farming occurs. For example, commercial broiler farms in Ecuador reported 100% prevalence of Eimeria spp., with Eimeria maxima (80.4%), Eimeria acervulina (70.6%), Eimeria praecox (55.4%), Eimeria tenella (53.6%), Eimeria necatrix (52.2%), and Eimeria brunetti (30.8%) [14]. In Vojvodina, Serbia, the prevalence of E. acervulina (37%), E. maxima (17%), E. mitis (25%), and E. tenella (48%) was reported in broilers [15]. In Africa, multiple Eimeria species were detected on 63% of farms, with up to six species on a single farm, including 100% prevalence in Ghana, 94% in Tanzania, and 77% in Zambia [16].

Notably, disease prevalence and species composition vary across production systems. Intensive broiler operations, layer systems, and backyard or village poultry differ in stocking density, hygiene, litter management, and exposure risk, thereby influencing infection pressure and transmission dynamics. Studies have demonstrated distinct Eimeria occurrence across different farm types and rearing systems, with a higher risk in large-scale broiler farms than in other systems [17].

The severity of infection depends on the infecting Eimeria species [18]. Eimeria brunetti, E. maxima, E. necatrix, and E. tenella are highly pathogenic and are associated with hemorrhagic coccidiosis, resulting in high morbidity and mortality [17]. In contrast, E. acervulina exhibits moderate pathogenicity, whereas E. mitis and E. praecox are considered less pathogenic, typically causing mild infections [19, 20].

Coccidiosis is transmitted through direct or indirect contact with feces from infected birds [11]. Infection occurs when birds ingest sporulated oocysts present in contaminated feed, water, or litter [21]. Although coccidiosis can affect chickens of any age, younger birds are more susceptible due to their immature immune systems. Each Eimeria species exhibits tissue specificity within the gastrointestinal tract. For example, E. tenella primarily infects the caeca, E. brunetti the ileum and rectum, E. necatrix the jejunum and caeca, and E. maxima the duodenum, jejunum, and ileum. Additionally, E. zaria, E. nagambie, and E. lata inhabit the duodenum and ileum, whereas E. praecox, E. mitis, and E. acervulina primarily infect the duodenum and jejunum [22, 23]. The disease typically affects the intestine within approximately 5 days and the caecum within 6 days following infection [24].

Eimeria infections disrupt gut homeostasis, leading to reduced feed intake, impaired digestion, malabsorption, and decreased weight gain [25]. These pathological effects are influenced by host-related factors, such as nutritional status, gut microbiota composition, environmental stressors, and concurrent infections with bacterial or viral pathogens. These factors interact with parasite genetic diversity to determine disease severity and host susceptibility. Infections may involve single or multiple Eimeria species simultaneously [26]. Poor husbandry practices, high stocking density, low-quality litter, and high humidity further increase the risk of infection [27].

Despite advancements in management and control strategies, coccidiosis remains a major threat to poultry health and productivity. In addition to clinical and diagnostic challenges, genetic variation within Eimeria spp. plays a critical role in shaping disease dynamics. The interaction between parasite genetic diversity and host-related factors, including nutrition, microbiota, and production systems, ultimately determines disease outcomes and the success of control. Understanding these genetic variations is essential for improving diagnostic accuracy, evaluating virulence, and managing drug sensitivity, thereby bridging conventional knowledge of Eimeria spp. with emerging genomic insights to support more effective control strategies.

## GENETIC MUTATIONS IN CHICKEN *EIMERIA SPP*.

Genetic mutations such as SNPs, CNVs, insertions/deletions, and structural variations introduce key sources of diversity among *Eimeria* spp. The identification of these mutations provides insights into unknown biological functions, evolutionary mechanisms, and opportunities for improving diagnostic approaches. To consolidate current knowledge, key mutations, affected genes, and associated phenotypic outcomes are summarized in Table 1 [28–32].

**Table 1:** Summary of key genetic variations in *Eimeria* spp. and associated phenotypic outcomes.

Type of genetic mutation	Chromosome/locus	Gene(s) affected	Phenotypic outcome reported
SNP	Chromosome 11	FAM96B	Gastrointestinal traits, digestive system development, potential resistance to infection [28]
SNP	Chromosome 11	RRAD	Metabolic traits, body weight gain, host resilience [28]
SNP (genome-wide)	Multiple	Multiple (non-synonymous)	Drug resistance, antigenic variation, host–pathogen interactions [29]
CNV	Multiple	Unknown/intergenic	Potential impact on adaptation and genome plasticity [29]
Structural variations (SVs)	Multiple	Multiple	Rapid adaptation and genomic heterogeneity [29]
Indel	Multiple	Multiple	Modulation of coding and non-coding regions, potential effects on virulence [29]
ITS-1 nucleotide variation	ITS-1 region	Not applicable	Increased intra-species diversity; diagnostic challenges [30, 31]
Epigenetic regulation	Genome-wide	Histone-modifying genes, non-coding RNAs	Stage-specific gene expression and modulation of virulence and drug response [32]

Beyond single-point mutations, broader genomic variations contribute to *Eimeria* adaptation and host–parasite interactions. SNPs have been associated not only with drug resistance but also with host performance traits such as body weight gain and immune response following infection. Notably, SNPs on chromosome 11 within FAM96B and RRAD have been linked to gastrointestinal and metabolic traits, potentially influencing host resilience [28].

Comparative genomic analyses highlight the complexity of variation in *E. tenella*. Genome-wide studies have identified approximately 46,888 SNPs, 15,107 insertions/deletions, 1,693 structural variants, and 3,578 CNVs [29]. Most polymorphisms are concentrated in intergenic and regulatory regions, whereas protein-coding and splice-site regions are relatively conserved. This uneven distribution underscores the genomic heterogeneity and adaptive capacity of *Eimeria* spp. Sexual reproduction and meiotic recombination further enhance genetic diversity in field populations, accelerating the emergence of advantageous variants [33].

Genetic variation in *Eimeria* spp. is shaped by mutations such as SNPs, CNVs, insertions/deletions, and inversions. Similar evolutionary processes have been observed in other apicomplexan parasites, including *Plasmodium* and *Toxoplasma*, where recombination, gene flow, and selective pressures drive population structure and trait evolution. Although less studied in *Eimeria*, emerging evidence suggests that recombination and mixed infections in poultry systems contribute significantly to genetic diversity and adaptation [32].

Key virulence-associated proteins include surface antigens (SAGs), apical membrane antigen-1 (AMA1), microneme proteins (MICs), and rhoptry proteins . Despite sequence variability, these mutations often converge functionally to influence host cell invasion, immune evasion, and metabolic adaptation. For example, polymorphisms in AMA1 and MICs can alter invasion mechanisms, while variation in SAGs affects immune recognition, and changes in rhoptry proteins influence intracellular survival. This functional convergence provides a clearer understanding of how genetic diversity translates into phenotypic outcomes relevant to virulence, vaccine efficacy, and drug sensitivity [8].

Genetic diversity also affects host–parasite interactions, as certain chicken breeds exhibit greater tolerance to infection [28]. Variations in the ITS-1 region enable species identification through ITS-based primers; however, nucleotide polymorphisms within this region increase intra-species diversity and complicate diagnostics [30, 31, 34].

Genetic variation in chicken-infecting *Eimeria* spp. represents a dynamic evolutionary system shaped by multiple selective pressures, including host immunity, vaccination, anticoccidial drug use, and intensive production practices [33, 35]. These pressures drive allele turnover and population diversification. Vaccine-induced and naturally acquired immunity may select for antigenic variants capable of immune evasion, while sustained drug pressure promotes mutations in drug-target genes [9]. High stocking density and repeated parasite cycling further accelerate the spread of adaptive variants [21, 37].

A major challenge across vaccine efficacy, diagnostic reliability, and drug sensitivity is the presence of cryptic genotypes or operational taxonomic units (OTUs) within *Eimeria* spp. [35]. These genetically distinct yet morphologically indistinguishable lineages often evade detection by conventional diagnostics and may not be represented in vaccine strains or drug sensitivity assays [10]. Consequently, cryptic OTUs contribute to vaccine failure, inconsistent treatment outcomes, and regional variability in disease control [37].

Beyond DNA-level variation, epigenetic mechanisms such as histone modifications, chromatin remodeling, and non-coding RNAs may regulate stage-specific gene expression and phenotypic plasticity in *Eimeria* spp. [38]. This emerging layer of regulation may influence virulence, drug response, and immune evasion, further complicating disease control and increasing the likelihood of diagnostic inaccuracies due to genetic variation and primer mismatches.

## VIRULENCE

Genetic variation within chicken-infecting *Eimeria* spp. populations play a critical role in determining phenotypic diversity, including differences in pathogenicity and infection severity. Molecular surveys and comparative sequencing studies have revealed the presence of intraspecific and cryptic genotypes across diverse geographic regions and production systems, indicating that virulence is not a fixed trait but rather a variable and heritable characteristic shaped by parasite population structure [39]. Variation in virulence has been directly linked to measurable disease outcomes *in vivo*, including differences in lesion severity scores, oocyst output, weight gain depression, and feed conversion efficiency, thereby establishing a direct connection between parasite genetic diversity and production losses under commercial conditions [28].

Variants implicated in virulence include SNPs, insertions/deletions, CNVs, and larger SVs identified through chromosomal-scale assemblies [29]. Genome comparisons of *E. tenella* and other chicken-infecting *Eimeria* spp. at both population and isolate levels have demonstrated that genome-wide polymorphisms and locus-specific diversity within gene families influence host–parasite interactions through surface antigens, secreted proteins, and rhoptry proteins [32]. These variations provide a substrate for natural and drug-induced selection.

A consistent observation is the enrichment of polymorphisms in genes encoding surface antigens (SAGs), microneme proteins (MICs), and rhoptry proteins (ROPs), including apical membrane antigen-1 (AMA1) and rhoptry neck (RON) proteins. These molecules are key mediators of virulence as they regulate host cell invasion, immune recognition, and modulation of host responses, including interference with cytokine signaling and innate immune pathways [40, 41]. Functional studies have demonstrated that AMA1 and RON proteins are involved in host cell invasion, whereas rhoptry kinases and other secreted effectors regulate intracellular survival and host pathology [42]. Consequently, allelic variation at these loci can influence invasion efficiency, parasite replication, and the extent of intestinal damage, as reflected in lesion severity and overall disease outcome in experimental infections [43].

Forward genetic and experimental evolutionary studies have further demonstrated that alterations in the genetic composition of *Eimeria* spp. directly influence drug susceptibility and fitness-related traits [44]. Selection experiments combined with linkage-group analyses have identified genomic regions and candidate mutations associated with resistance to anticoccidials such as monensin and diclazuril, which may also affect parasite growth and pathogenicity *in vivo* [9]. These findings highlight that selection pressures can rapidly alter allele frequencies within *Eimeria* populations and that mutations may exert pleiotropic effects on virulence.

Comparative infection studies, together with transcriptomic and proteomic profiling of high- and low-virulence isolates, have linked allelic variation, differential gene expression, and gene presence or absence to differences in tissue tropism, lesion severity, and fecundity [45]. For instance, isolate-specific variation in microneme and rhoptry repertoires, as well as surface antigen expression, has been associated with differences in invasion mechanisms and immune evasion strategies, ultimately contributing to distinct virulence phenotypes [29].

Despite these advances, many genotype–phenotype associations remain inferred from comparative genomics and omics-based analyses, with relatively few mutations functionally validated through targeted gene disruption or controlled *in vivo* models. This represents a significant gap between genomic predictions and experimentally confirmed virulence determinants.

Genetic polymorphisms in virulence-associated genes also complicate advances in diagnostics and vaccine development. In diagnostics, primer or probe mismatches may arise in molecular assays, whereas in vaccines, antigenic divergence may reduce cross-protection among heterologous strains [33, 46]. Furthermore, rapid allele evolution under drug pressure suggests that resistance and virulence traits may emerge or shift under intensive management conditions [47]. Therefore, integrated strategies, including genomic surveillance of field populations, development of vaccines targeting conserved or multivalent antigens, and optimized anticoccidial management practices, are essential.

Understanding how genetic mutations influence *Eimeria* spp. provides critical insight into parasite infectivity. However, it is equally important to evaluate how these mutations influence host immune responses. Variations in genes encoding surface antigens, secreted proteins, and invasion-associated molecules can alter immunogenicity, disrupt cytokine signaling pathways, and interfere with the development of protective immunity. These changes may compromise vaccine-induced responses or facilitate immune evasion. Therefore, alongside virulence, immunogenicity represents a key determinant of host resistance and vaccine success.

## IMMUNOGENICITY

Genetic variation in *Eimeria* spp. directly influences antigenicity and host immune responses. Antigenic differences between isolates can reduce cross-protective immunity and complicate vaccine development, while stage-specific and isolate-specific expression of surface and secreted proteins determines antigen presentation to the host immune system [46, 48, 49]. Genetically distinct *Eimeria* strains can bias host immune responses toward either humoral or cell-mediated pathways, although comprehensive comparisons across genotypes remain limited [50].

Early cross-protection studies demonstrated that immunization with a single *Eimeria* isolate often provides incomplete protection against heterologous isolates, highlighting substantial antigenic diversity in species such as *Eimeria maxima* and *Eimeria* *tenella* [51, 52]. These findings illustrate how antigenic divergence undermines the efficacy of single-strain vaccines [46]. More recent population genomic and antigen-focused studies have confirmed extensive haplotype and sequence variation in SAGs and apical proteins such as AMA1 and immune mapped protein-1 (IMP1), further contributing to reduced cross-protection when vaccines target highly polymorphic loci [53].

Variation in genes encoding glycosylphosphatidylinositol-anchored surface antigens, MICs, AMA/RON complexes, and other secreted effectors leads to changes in epitope structure and antigen abundance, influencing B-cell and T-cell recognition [54, 55]. These parasite-driven effects occur alongside host genetic diversity, where polymorphisms in major histocompatibility complex (MHC) alleles and immune-related genes regulate antigen presentation and T-cell activation, ultimately shaping strain-specific immune responses.

Comparative genomic and functional studies, including transgenic expression of AMA1 and IMP1 in *E. tenella*, have demonstrated that antigen manipulation can significantly alter protective immune responses in chickens [53]. Transcriptomic and proteomic comparisons between virulent and attenuated isolates further reveal differences in the expression of invasion-related and surface proteins that influence antigen exposure [45, 56]. For example, RNA sequencing analysis of precocious and parental *E. maxima* lines demonstrated altered expression of invasion-associated genes, correlating with reduced fecundity while maintaining immunogenicity [57]. Similarly, transcriptional profiling of *E. tenella* sporozoites revealed distinct expression patterns between virulent and attenuated strains [45].

These findings indicate that both allelic variation and differences in gene expression contribute to altered immunogenicity. Such variation also influences the balance between humoral and cell-mediated immunity, particularly interferon-γ-mediated T-cell responses, which are essential for protection against intracellular stages.

Because many immunodominant vaccine targets are highly polymorphic, vaccines based on single antigens or single isolates often fail to provide broad protection [58]. This limitation has driven the development of multivalent live vaccines incorporating multiple isolates, as well as strategies that target conserved antigens or use antigen cocktails [59, 60]. However, sustained vaccine pressure may select for antigenic variants capable of immune escape, leading to shifts in allele frequencies within field populations. Additionally, sequence variation in immunogenic loci may affect the performance of molecular diagnostics and antigen-based serological assays if variable regions are targeted [61].

Overall, genetic variation in *Eimeria* spp. significantly influences immunogenicity by altering antigen recognition and immune response dynamics. Variations in immunodominant loci affect epitope presentation and immune recognition, thereby influencing the strength and durability of protective immunity. When combined with host genetic variability, these parasite-driven changes have profound implications for vaccine efficacy, diagnostic reliability, and the long-term control of coccidiosis.

## VACCINE EFFICACY

Chicken-infecting *Eimeria* spp. exhibit high levels of genetic diversity, which ultimately has implications for host immunity and vaccine design. Current vaccine development consists mainly of live attenuated or wild-type parasites, which often confer partial or strain-specific protection. Therefore, understanding the genetic factors underlying antigenic diversity is essential for improving vaccine efficacy and sustainability in poultry health management [33, 48]. To conceptualize these challenges across vaccine platforms, a comparative summary of currently employed and emerging vaccine types, including live wild-type, live attenuated, recombinant subunit, multiepitope, and vectored vaccines, is presented in Table 2 [33, 35, 39, 48, 62–67].

**Table 2:** Comparison of vaccines available for the control of chicken coccidiosis.

Vaccine	Key characteristics	Advantages	Limitations and challenges
Live wild-type	Composed of mixed populations of naturally occurring *Eimeria* strains administered at low doses	Broad antigen exposure, relatively low cost, scalable via oocyst production	Strain-specific and species-specific immunity, variable field performance due to antigenic mismatch with circulating isolates, risk of residual pathogenicity and genetic drift [33, 62, 63]
Live attenuated	Attenuated strains generated through serial passage or selection under defined pressures	Reduced virulence with retained immunogenicity, widely used commercially	Genetic drift, gene loss, or altered antigen expression during attenuation, inconsistent protection against heterologous field strains, updating requires redevelopment and revalidation [35, 64, 65]
Recombinant subunit	One or a few defined antigens, such as AMA1, MICs, and SAGs, expressed in heterologous systems	High antigenic precision, genetic stability, flexible antigen selection	Limited antigenic breadth, often strong laboratory immunogenicity but limited field validation, higher production costs and reliance on adjuvants [48, 66, 67]
Multiepitope	Constructs designed *in silico* by incorporating multiple conserved B-cell and T-cell epitopes	Potential for broader cross-protection, adaptable to emerging strains	Predominantly experimental, limited *in vivo* and field data, delivery and expression challenges, high development costs [48, 67]
Vectored	*Eimeria* antigens delivered via viral or bacterial vectors	Enhanced cellular immunity, high flexibility for antigen updates	Regulatory complexity, higher production costs, limited commercial-scale field data, mass-administration challenges [39, 67]

The highly dynamic nature of *Eimeria* genomes, driven by the accumulation of mutations in fundamental loci such as AMA1, SAGs, and MICs, can alter protein structure, antigenicity, and epitope recognition, thereby reducing vaccine-induced immunity. These changes affect vaccine-induced antibodies, limiting their ability to recognize and neutralize antigenically divergent strains effectively. Furthermore, protective immunity that develops after coccidial infection is highly species- or strain-specific [63]. This indicates that the high degree of antigenic specificity in existing vaccines may not confer protection in hosts infected with antigenically divergent or cryptic genotypes [67]. Whole-genome sequencing has identified gene duplications and deletions that further diversify antigens within and between populations [32].

Due to species-specific vaccination and the development of species-specific immunity, acquired immunity in host species is sometimes strain-restricted [63]. Antigenic mismatch may occur when birds vaccinated with commercial vaccines designed from a few reference strains are exposed to antigenically divergent field isolates or OTUs, resulting in breakthrough infections even in immunized flocks [68]. Thus, strain-specific protection has limitations regarding cross-protection against heterologous species. The presence of OTUs may further limit cross-protection, as vaccine strains may not encompass the full spectrum of genetically distinct lineages circulating in different production systems.

Attenuated vaccines generated through serial passage or targeted selection of *Eimeria* strains under specific environmental or host pressures are designed to reduce parasite virulence while preserving immunogenicity. However, this process can inadvertently induce genetic drift, gene loss, or epigenetic alterations that affect crucial virulence and antigen expression pathways [35, 64]. These changes may influence the expression of immunodominant surface and secretory proteins, including AMA1, MICs, and SAGs, which are essential for eliciting effective host immune responses [66]. Therefore, attenuated strains may exhibit altered antigenic expression compared with wild-type vaccines, potentially decreasing the robustness of induced immunity [65].

Studies on *Eimeria maxima* represent a well-studied example in which cross-immunity between isolates is often incomplete, with variable levels of protection depending on the infecting parasite strain and host genetic background [46]. This indicates that *Eimeria* infections are largely strain-dependent. In experimental infection studies, protection against homologous strains is generally robust, whereas exposure to heterologous strains results in reduced performance parameters, such as body weight gain, lesion scores, and oocyst output [52, 62]. Such antigenic specificity explains the inconsistent performance of commercial vaccines across different geographic regions, where circulating *Eimeria* populations may contain genotypes or OTUs not included in vaccine formulations [39, 67]. Furthermore, discrepancies are frequently observed between laboratory efficacy trials and field performance of coccidiosis vaccines, as controlled experimental challenges typically involve homologous reference strains under standardized conditions that do not fully capture the genetic complexity, mixed-species infections, and environmental pressures present in commercial poultry systems [65]. As a result, vaccines demonstrating robust protection in experimental studies may exhibit variable or reduced effectiveness under field conditions.

In light of these challenges, the development of next-generation vaccines against coccidiosis requires a deeper understanding of the genomic landscape and population structure of *Eimeria* spp. The formulation of multiepitope or multivalent vaccines that incorporate conserved antigens from multiple *Eimeria* spp. and strains represents one strategy to mitigate antigenic mismatch. Whole-genome sequencing of field isolates, together with immunoproteomic analysis, may be essential for identifying conserved immunogenic targets less prone to diversification. Furthermore, integrating host genetics, particularly MHC haplotypes, into vaccine efficacy studies may help explain variable immune outcomes across chicken breeds [69]. Continuous surveillance of *Eimeria* populations and adaptive updating of vaccine strains, analogous to influenza vaccine strategies, may enhance long-term control of chicken coccidiosis. However, the feasibility of adaptive vaccine strategies in commercial poultry production is influenced by cost, scalability, and regulatory constraints. While live and attenuated vaccines remain relatively cost-effective and scalable, updating these vaccines is logistically complex [65], whereas recombinant, multiepitope, and vectored vaccines offer greater flexibility for antigen updates but are currently associated with higher production costs and challenges in large-scale delivery.

Understanding the influence of genetic variation on vaccine efficacy highlights the dynamic evolutionary relationship between *Eimeria* populations and host immune defenses. The emergence of antigenically diverse or cryptic genotypes has revealed that control strategies based solely on immunoprophylaxis must contend with a constantly shifting genetic landscape. However, the impact of genetic variation extends beyond host–parasite interactions and plays a major role in parasite susceptibility to anticoccidial drugs, shifting patterns of resistance under selective pressure. Therefore, understanding the mechanisms through which genomic variation in *Eimeria* spp. alters drug sensitivity is particularly important given the widespread use of chemoprophylaxis in poultry production and the growing concern of resistance. Collectively, these considerations highlight the importance of integrating genetic diversity, field-level vaccine performance, and commercial feasibility when developing sustainable coccidiosis control strategies.

## DRUG SENSITIVITY

Avian coccidiosis remains a serious concern in the poultry industry worldwide, prompting the implementation of numerous control techniques, including synthetic compounds and anticoccidial supplements. However, the emergence of altered drug sensitivity and resistance due to the widespread and prolonged use of anticoccidial drugs, together with the presence of genetic variants within *Eimeria* spp., has increased the complexity of coccidiosis control and prevention. As drug resistance and altered sensitivity among *Eimeria* spp. continue to increase worldwide, the efficacy of available anticoccidial drugs has declined [70]. Historically, resistance to anticoccidial drugs has emerged sequentially following their introduction, with early resistance reported against sulfonamides and amprolium shortly after their widespread use in the mid-20th century. This was followed by resistance to ionophores such as monensin, salinomycin, and maduramycin, and more recently to synthetic chemicals, including diclazuril, toltrazuril, and decoquinate. This progressive timeline reflects sustained selective pressure imposed by long-term chemoprophylaxis and underscores the cumulative erosion of efficacy across successive anticoccidial classes [36, 71].

The development of altered drug sensitivity in *Eimeria* spp. has been closely linked to diverse genomic mutations, with different resistance mechanisms identified depending on the anticoccidial drug involved. For instance, whole-genome sequencing of decoquinate-resistant *Eimeria tenella* strains revealed non-synonymous mutations in the cytochrome b gene, including novel substitutions such as Gln131Lys, Phe263Leu, and Phe283Leu, located in the extracellular binding pocket, strongly associating mutant alleles of this gene with resistance to decoquinate [72]. In contrast, resistance to salinomycin has been attributed to a single-point mutation (T611C) in the adrenodoxin oxidoreductase gene (EtADR), resulting in the amino acid substitution F204S and reduced susceptibility to the drug [47]. These examples highlight how specific single-gene mutations, whether in mitochondrial or nuclear-encoded proteins, can confer resistance to different anticoccidials. Resistance-associated mutations may impose fitness costs on *Eimeria* spp. in the absence of drug pressure, potentially reducing parasite replication rate, competitive ability, or transmission efficiency relative to drug-sensitive strains. Such fitness trade-offs may facilitate partial reversion to drug sensitivity following withdrawal or rotation of anticoccidials, although compensatory mutations can offset these costs, allowing resistant genotypes to persist in commercial poultry systems [36].

Drug sensitivity in *Eimeria* spp. has been evaluated using six anticoccidial agents, namely clopidol, diclazuril, maduramycin, monensin, salinomycin, and toltrazuril, through standard assessment indices such as the anticoccidial index, percentage of optimum anticoccidial activity, relative oocyst production, and reduction in lesion scores. The results indicated that all field isolates exhibited severe resistance to the tested anticoccidials, particularly to diclazuril, maduramycin, and toltrazuril [70].

Although chemical anticoccidials and ionophores negatively affect parasite metabolism and ion transport [3], some anticoccidial drugs are less effective during specific developmental stages of *Eimeria* spp. [73]. Changes in the biochemical composition of the *Eimeria* membrane have contributed to the development of ionophore resistance [74]. In response to escalating drug resistance, increasing attention has been directed toward non-chemical alternatives to chemoprophylaxis, including phytogenic compounds, probiotics, prebiotics, and immunomodulators. These strategies aim to enhance gut health, modulate host immune responses, and limit parasite replication without imposing strong selective pressure on *Eimeria* populations. Such alternatives are increasingly investigated as complementary or integrative approaches alongside vaccination and improved management practices [75].

Because *E. tenella* is one of the most prevalent infecting species and has a substantial impact on the poultry industry [17], drug resistance in this species has been investigated using standard assessment methods. The results indicated severe resistance to salinomycin and nicarbazine, moderate resistance to amprolium and clopidol, slight resistance to toltrazuril, and sensitivity to sulfachloropyrazine sodium [76].

Furthermore, the A1852G mutation in the cytoplasmic prolyl-tRNA synthetase gene (EtcPRS) has been shown to impair halofuginone binding, thereby reducing *E. tenella* susceptibility to this drug. Using forward and reverse genetic approaches, a molecular marker associated with halofuginone resistance was identified, and whole-genome sequencing revealed point mutations in ETH2_1020900, which encodes prolyl-tRNA synthetase. Introduction of these mutations into *E. tenella* validated the gene overexpression, confirming resistance in both *in vivo* and *in vitro* settings. Collectively, these findings suggest that A1852G and A1854G mutations in ETH2_1020900 are key contributors to halofuginone resistance in *E. tenella* [77].

In addition, the enolase-2 protein of *E. tenella* (EtENO2) has been found to be differentially expressed in drug-sensitive and drug-resistant strains, with higher expression observed in strains resistant to diclazuril, maduramycin, and salinomycin. Quantitative real-time PCR and western blot analyses revealed upregulation of EtENO2 transcription in field-isolated resistant strains compared with drug-sensitive counterparts, accompanied by increased catalytic activity. Expression patterns also varied across developmental stages, with second-generation merozoites and unsporulated oocysts showing higher EtENO2 levels than sporozoites and sporulated oocysts. Immunofluorescence assays demonstrated its distribution throughout sporozoites and second-generation merozoites, including on their surfaces and localized to the parasitophorous vacuole membrane following sporozoite invasion. Secretion assays further confirmed that EtENO2 can be released outside sporozoites. These findings suggest a role for EtENO2 in host–parasite interactions and its potential involvement in resistance to certain anticoccidial drugs [78].

The mechanisms underlying drug sensitivity in *Eimeria* spp. to many available anticoccidial drugs remain relatively unclear. Experimental evolution, linkage-group selection, and pooled genome sequencing have shown promising results. These strategies have been used to locate genomic loci potentially responsible for the sensitivity of *E. tenella* to monensin and diclazuril. The study focused on two *E. tenella* strains with different drug sensitivity backgrounds that were crossed under specific selection pressure applied to uncloned progeny, producing a population resistant to both drugs [9].

Forward genetic mapping has been employed to identify genetic loci in *Eimeria* spp. associated with strain-specific immunity and drug sensitivity [79]. Despite its usefulness, this approach presents several limitations [9], including the requirement for cloned strains with a clear genetic background, which necessitates labor-intensive laboratory procedures. The generation of drug-resistant *Eimeria* through experimental evolution also involves repeated *in vivo* passages over multiple generations, making the process highly time-consuming [80]. Moreover, as *Eimeria* undergoes both sexual and asexual development, the higher mutation rate observed during sexual reproduction accelerates the emergence of drug-resistant strains, although this is often accompanied by numerous non-specific mutations [9, 81].

Drug sensitivity and resistance remain growing concerns in the control of chicken coccidiosis. In addition to the mechanisms of drug resistance mentioned above, the continuous evolution of chicken-infecting *Eimeria* spp. through accumulating genetic mutations and variations has further complicated the control, diagnosis, and treatment of coccidiosis. Variation in drug sensitivity observed among field isolates may partly reflect the circulation of cryptic OTUs with distinct resistance-associated genotypes that are not captured by standard susceptibility testing. Therefore, investigating the genetic diversity of *Eimeria* spp. is essential not only for monitoring drug sensitivity but also for refining and validating diagnostic methods that accurately detect diverse *Eimeria* spp. Mutations conferring resistance can co-occur with changes in surface antigens or secretory proteins, linking drug sensitivity, vaccine escape, and diagnostic reliability within a network of genotype-driven phenotypic outcomes. These findings emphasize that sustainable coccidiosis control requires integrated strategies combining informed anticoccidial use, resistance surveillance, non-chemical interventions, and vaccination to mitigate the evolutionary consequences of prolonged chemoprophylaxis.

## DIAGNOSTIC MECHANISMS

The accumulation of genetic diversity within and between *Eimeria* spp. can significantly reduce the sensitivity, specificity, and reliability of existing and future diagnostic mechanisms. The presence of polymorphisms, paralogous copies, OTUs, and antigenic variation can create inconsistencies among diagnostic targets, such as primers, probes, antigens, and circulating parasite species. This results in false negatives, incorrect identification, and misleading prevalence estimates if assays are not validated across geographic and genetic variation [33].

Although traditional oocyst morphology and lesion scoring remain useful diagnostic techniques, they have limitations, including morphological overlap among species, mixed infections that obscure features of less abundant species, and the presence of OTUs that are morphologically indistinguishable from described species [82]. Thus, morphology-based diagnosis alone may misrepresent species composition and prevalence when genetic diversity and mixed infections are present. Comparative evaluations indicate substantial differences in diagnostic performance across methodologies, with conventional morphology-based identification generally exhibiting lower sensitivity and specificity than molecular approaches, particularly in mixed infections. PCR and qPCR assays typically demonstrate higher sensitivity and species-level specificity than morphology and serology, while qPCR further enables quantitative assessment of parasite burden [83]. However, next-generation sequencing (NGS) provides the highest resolution for detecting low-abundance species, mixed infections, and cryptic genotypes, although at increased cost and analytical complexity [84].

Furthermore, serological tests and antigen-capture enzyme-linked immunosorbent assays (ELISAs) depend on conserved immunodominant proteins. The accumulation of antigenic polymorphisms, differential expression between strains, and life-stage-specific antigens may reduce sensitivity and lead to cross-reactivity between species [46]. Although some conserved antigens, such as 3-1E and other common immunodominant proteins, could support broadly reactive assays, validation across *Eimeria* spp. is necessary because sequence and structural variations in surface antigens can alter antibody binding and affect test performance. Despite the superior analytical performance of molecular diagnostics, their deployment in low-resource or field settings remains constrained by infrastructure, cost, and technical expertise. Consequently, there is growing interest in simplified, field-deployable diagnostic platforms such as loop-mediated isothermal amplification, lateral flow assays, and portable qPCR systems. These platforms offer reduced equipment requirements, rapid turnaround times, and potential applicability in resource-limited poultry production environments [85].

Most molecular assays currently used for *Eimeria* spp. rely on ribosomal loci such as ITS1, ITS2, and *18S rDNA* or other conserved genes [22]. The presence of intraspecific polymorphisms and paralogous ITS copies can cause overestimation of diversity or inconsistent amplification [86]. Furthermore, single-primer or probe sets may fail when template mismatches occur due to field genotypes, resulting in false negatives or biased quantification [31]. Many studies have demonstrated either the failure of established species-specific PCR assays to detect divergent OTUs or high ITS heterogeneity within single genomes, underscoring the limitations of unvalidated single-locus assays [39, 68]. A further challenge is the lack of standardized diagnostic protocols, reference materials, and reporting thresholds across laboratories and countries, which complicates the comparison of prevalence data and resistance surveillance at regional and global scales. Variability in DNA extraction methods, primer design, amplification conditions, and bioinformatic pipelines can lead to inconsistent results, underscoring the need for harmonized guidelines, inter-laboratory validation, and standardized reference panels for *Eimeria* diagnostics [87]. OTUs further complicate diagnostics by evading detection with standard PCR or antigen-based assays, leading to underestimation of infection prevalence and misidentification of circulating species [68]. Newer approaches, such as multiplex qPCR or NGS, could reduce certain risks but would still require validation, as transcript-based assays may vary across strains and life stages [10].

Diagnostic assays such as PCRs, species-specific primers or probes, and ELISAs should therefore be validated against geographically and genetically diverse species to detect primer mismatches or antigenic variation. Combining independent genetic targets, such as nuclear *rDNA* or multiple antigen targets in multiplex PCR assays, reduces the likelihood that variation at a single locus will produce incorrect results. Furthermore, NGS may reveal cryptic diversity and mixed infections that single-locus assays miss, although bioinformatic pipelines should be designed to account for ITS paralogs and CNVs. Understanding diagnostic methods used for other apicomplexan parasites may improve diagnostic mechanisms for *Eimeria* spp. Diagnostic failures are not isolated phenomena; rather, they often reflect the combined impact of antigenic divergence, cryptic genotypes, and drug-resistant variants, emphasizing the need for integrated surveillance that simultaneously considers genomics, immune response, and therapeutic interventions.

## CONCLUSION

Genetic variation within chicken-infecting *Eimeria* spp. emerges as a central determinant of coccidiosis dynamics, influencing virulence, immunogenicity, vaccine efficacy, drug sensitivity, and diagnostic reliability. Across the reviewed evidence, polymorphisms such as SNPs, CNVs, insertions/deletions, and SVs were consistently associated with phenotypic diversity, including variation in lesion severity, oocyst output, host immune responses, and production performance. Variability in key antigenic proteins, including AMA1, SAGs, MICs, and ROPs, was shown to underpin differences in host cell invasion, immune evasion, and pathogenicity. Furthermore, genomic mutations in drug-target genes and in metabolic pathways were associated with reduced susceptibility and the emergence of resistance to commonly used anticoccidials. Diagnostic inconsistencies were also strongly associated with genetic heterogeneity, particularly in ribosomal loci such as ITS regions and *18S rDNA*, leading to false negatives, species misidentification, and underestimation of infection prevalence. Collectively, these findings demonstrate that genetic diversity is not an isolated feature but a unifying driver affecting multiple control domains simultaneously.

From a practical perspective, these findings have significant implications for poultry health management. Vaccine strategies based on limited strains or highly polymorphic antigens may provide inconsistent protection under field conditions, emphasizing the need for multivalent or conserved antigen-based approaches. Similarly, the widespread emergence of drug resistance highlights the importance of rational anticoccidial use, including rotation programs, reduced reliance on chemoprophylaxis, and integration with non-chemical alternatives such as probiotics and phytogenic compounds. Diagnostic systems must also evolve toward multiplex, genomics-informed platforms that can accurately detect diverse and mixed *Eimeria* infections. Importantly, integrating genomic surveillance into routine disease monitoring can enable early detection of emerging virulent or drug-resistant strains, thereby supporting timely and evidence-based interventions.

A major strength of this review lies in its integrative approach, synthesizing evidence across traditionally separated domains, including virulence, immunogenicity, vaccination, drug resistance, and diagnostics. By linking genetic variation to functional and applied outcomes, this review provides a comprehensive framework for understanding the multifactorial nature of coccidiosis. Additionally, the inclusion of both molecular and applied studies enhances the translational relevance of the findings for field-level applications.

However, several limitations should be acknowledged. Much of the current understanding of genotype–phenotype relationships in *Eimeria* spp. is derived from comparative genomics and correlational studies, with relatively few mutations validated through controlled *in vivo* or *in vitro* experiments. Variability in study design, host systems, and analytical methodologies also limits direct comparison across studies. Furthermore, there is a geographic bias in the available data, with limited representation from low- and middle-income regions, where the coccidiosis burden is often highest. The lack of standardized diagnostic protocols and genomic reference frameworks further complicates the interpretation and application of findings.

Future research should prioritize functional validation of candidate genes associated with virulence, immunogenicity, and drug resistance using targeted genetic and *in vivo* infection models. The identification of conserved antigenic targets for next-generation vaccines, coupled with immunogenomic studies incorporating host factors such as MHC diversity, will be essential for improving vaccine performance. Advances in high-throughput sequencing and bioinformatics should be leveraged to establish global genomic surveillance systems capable of tracking evolving *Eimeria* populations. Additionally, the development of cost-effective, field-deployable diagnostic tools, such as multiplex qPCR and isothermal amplification platforms, will be critical for improving disease detection in resource-limited settings. Integrative strategies combining vaccination, optimized drug use, improved management practices, and non-chemical interventions should be further explored within a One Health framework.

In conclusion, genetic diversity within *Eimeria* spp. represents a dynamic and evolving challenge that underpins variability in disease expression, control efficacy, and diagnostic performance. Sustainable control of coccidiosis will require a coordinated, genomics-informed approach that integrates advances in molecular biology, immunology, epidemiology, and field management. By aligning research, surveillance, and practical interventions, it is possible to develop more resilient and adaptive strategies to mitigate the global impact of coccidiosis on poultry production systems.

## DATA AVAILABILITY

The data generated during the study are included in the manuscript.

## AUTHORS’ CONTRIBUTIONS

NMR: Writing – original draft and implementation. MAA: Supervision, conceptualization, and review and editing. Both authors have read and approved the final version of the manuscript.
